# Secondary Aorto-Colonic Fistula: A Case Report and Literature Review of a Rare Complication after EVAR

**DOI:** 10.1155/2022/8412460

**Published:** 2022-12-08

**Authors:** Nattawadee Wiangphoem

**Affiliations:** Department of Surgery, Sunpasitthiprasong Hospital, Ubon Ratchathani, Thailand

## Abstract

*Background*: Aorto-enteric fistula (AEF) is a rare but fatal condition. The incidence of the overall AEF was approximately 0.36–2%, but the incidence of the aorto-colonic fistula was scarcely reported. A history of abdominal pain, fever, or gastrointestinal bleeding (GIB) in a patient with a history of aortic intervention should be highly suspected of this condition. This report describes a patient with lower GIB after an endovascular aneurysm repair (EVAR) for a symptomatic abdominal aortic aneurysm (AAA). *Case Presentation*: A 65-year-old man with a history of EVAR for symptomatic AAA presented with a massive lower GIB for two weeks. He also had a history of left lower quadrant pain and low-grade fever. Diverticular disease was suspected, and medical treatment was administered. After the initial conservative treatment, a colonoscopy was performed. The findings showed a fistula that exposed an aortic stent graft at the left-sided colon. An aorto-colonic fistula was diagnosed. After administering intravenous (IV) antibiotics, a staged axillo-bifemoral bypass graft with aortic stent graft explantation was performed. The patient recovered well and was discharged home after a month of hospitalization and IV antibiotics. *Conclusion*: In a patient with a history of aortic intervention, any abdominal pain, unknown fever, or even GIB should be suspected of complications of aortic intervention. Highly suspicious of this rare condition is the key to an early diagnosis and prompt treatment.

## 1. Introduction

Secondary aorto-enteric fistula (AEF) is a rare condition. The incidence was reported to be approximately 0.036–1.6% [1]. A review and pooled data analysis, as discussed by Kakkos et al. [2], reported 823 patients from 216 publications. In this publication, only 6.3% of all patients developed secondary AEF after endovascular procedures. The most common site of the fistula was the duodenum, approximately 60%. Only 5% was identified in the large intestine.

Two-thirds of the patients would present with gastrointestinal bleeding (GIB), whereas the remaining would present with malaise and abdominal pain. The wide variety of clinical presentations in this rare condition makes early diagnosis and treatment difficult, leading to higher morbidity and mortality [3].

This study reports a case of secondary aorto-colonic fistula, followed by an endovascular procedure.

## 2. Case Presentation

**Figure 1:**
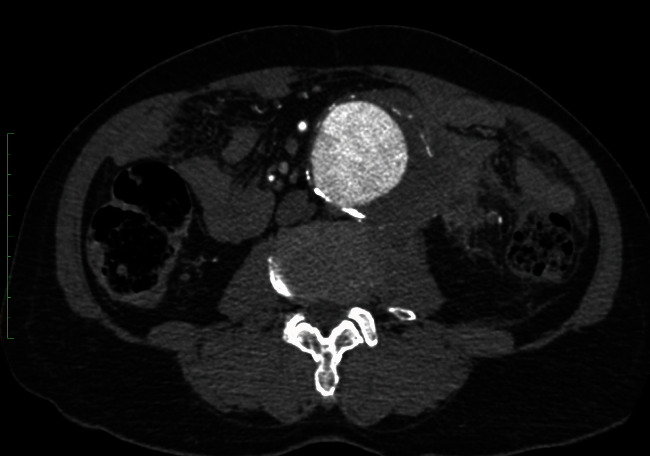
Preoperative CT scan revealed a large AAA with periaortic fat stranding.

A 65-year-old man with type II diabetes mellitus, hypertension, and chronic kidney disease presented with left paraumbilical pain radiated to the back for one week. He had no fever or other gastrointestinal symptoms. Physical examination revealed an 8 cm expansile mass with tenderness. The computed tomography angiography (CTA) showed infrarenal abdominal aortic aneurysm (AAA), 6.7 cm in size, with a periaortic fat stranding as shown in Figure 1.

Blood test showed hematocrit 37%, no leukocytosis, C-reactive protein (CRP) 84.6 mg/dL, and erythrocyte sedimentation rate (ESR) >130 mm/hour. Even if the ESR and CRP were rising, the patient had no history of fever before, so infected AAA was not suspected at that time. Endovascular aneurysm repair (EVAR) with Endurant II was immediately performed. After the operation, the patient had a low-grade fever with no specific symptoms. He was discharged home on postoperative day 3 with an improvement in abdominal pain. CTA at one month did not show any endoleaks. The patient was followed up at a three-month interval.

Six months after EVAR, he presented with vague abdominal pain and diarrhea, without fever. He went to another hospital and was diagnosed with diverticulitis. He was discharged home with an oral antibiotic.

Two weeks later, he returned to the same hospital with hematochezia and low-grade fever with stable vital signs. He received four units of packed red cells and was scheduled for an elective colonoscopy. The colonoscopic finding showed an aorto-colonic fistula at the sigmoid colon 30 cm from the anal verge. The patient was stabilized and referred to our hospital.

**Figure 2:**
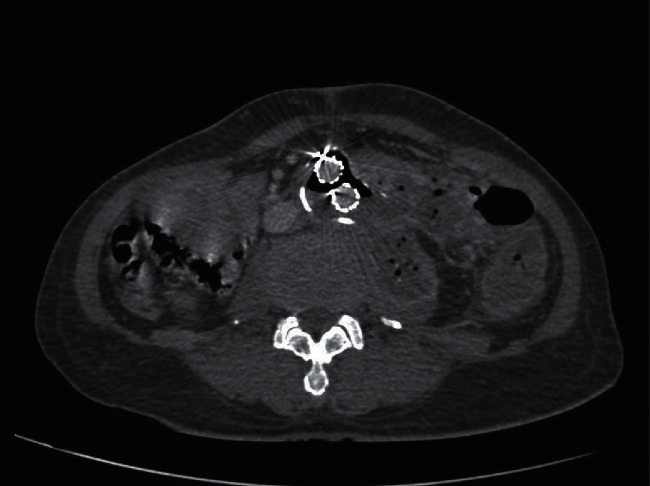
CTA revealed air in the aneurysm sac and left psoas abscess.

**Figure 3:**
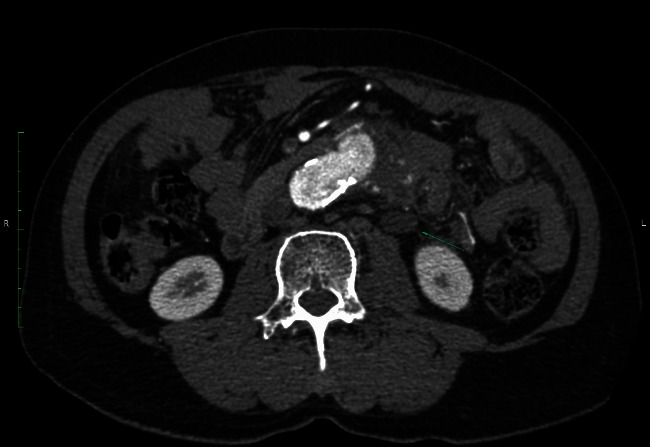
This slide showed the left hydronephrosis (arrow sign).

At first, the patient's vital signs were stable with no hematochezia. Laboratory test showed 22% hematocrit with leukocytosis (WBC 14,000 cells/mm^3^, N 86%), CRP 152.51 mg/dL, and ESR 58 mm/hour. Broad-spectrum intravenous (IV) antibiotics were started. CTA was immediately performed, and imaging demonstrated air in the aneurysm sac attached to the left-sided colon, left psoas abscess, and left hydronephrosis, as shown in Figures 2 and 3. No active contrast extravasation was observed. Due to his stable condition, a staged procedure was planned, the first stage with an axillo-bifemoral bypass graft followed by a second stage graft explantation and Hartmann's procedure.

An axillo-bifemoral bypass was performed two days after the complete preoperative evaluation. Before the second operation, a ureteric stent was inserted into the left ureter to prevent accidental injury during the open procedure. The second stage graft explantation and Hartmann's operation were performed a week later due to severe malnutrition.

**Figure 4:**
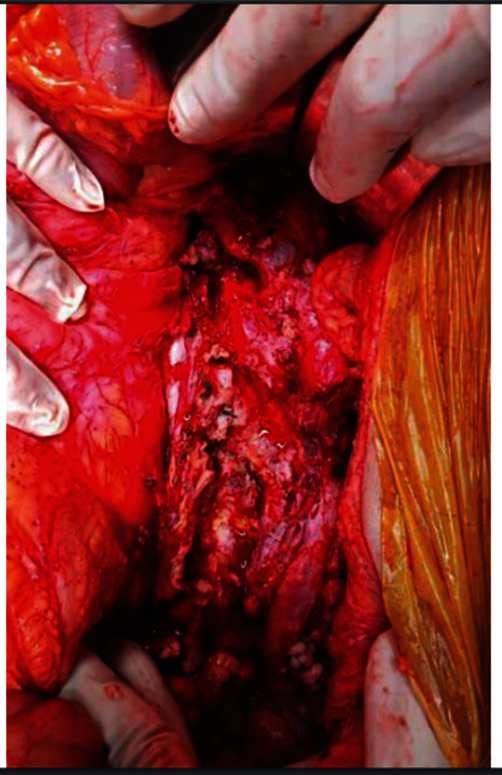
Most parts of the aneurysm sac were debrided.

After entering the abdominal cavity, the supraceliac aorta, suprarenal aorta, and both renal arteries were identified. The left hemicolectomy was performed first. The perforation site and the connection to the sac were identified. Then supraceliac aorta, both renal, external, and internal iliac arteries were controlled. The aneurysm sac was entered, and the stent grafts were identified and removed. Suprarenal hook was tried to remove from the proximal attachment using a 20-cc syringe but failed. All the fabric attached to the suprarenal hook was cut off, and the suprarenal hook was left inside. The supraceliac cross-clamp was then moved to the suprarenal cross-clamp. A proximal aortic stump was suture ligated with multiple layers of polypropylene 3-0. The distal common iliac arteries were suture ligated. Most of the aortic tissue was debrided, as shown in Figure 4. The abdomen was then irrigated with a large amount of normal saline solution. An omentum was used to wrap around the aortic stump.

The patient stayed in the intensive care unit for three days and returned to the regular ward. The culture of aortic tissue showed *Acinetobacter baumannii* (multidrug-resistant), *Klebsiella pneumoniae* (Carbapenem-resistant Enterobacteriaceae), and *Enterococcus faecium*. Pathological report of the aortic wall demonstrated an acute fibrinous arteritis with no signs of ischemic colitis. Proper parenteral antibiotics were administered for four weeks. The patient recovered well. An oral antibiotic was continued throughout the period. One month later, CTA revealed a small amount of fluid collection around the aortic stump and patented the axillo-bifemoral bypass graft. The laboratory finding showed unchanged levels of both ESR and CRP.

Eighteen months after the graft explantation, the patient's status was fully recovered with no signs of recurrent infection. CTA revealed complete resolution of all fluid collection near the aortic stump and the axillo-bifemoral bypass graft was still patented. ESR and CRP were decreased (121 mm/hour and 1.53 mg/dL, in order). The oral antibiotics was discontinued.

## 3. Discussion

Secondary AEF is a rare complication after an open aortic reconstruction. The etiologies are expected to be multifactorial, such as peri-anastomosis pseudoaneurysm formation at the suture line that created pressure necrosis in the bowel wall, intestinal trauma during dissection, direct pressure by pulsatile graft, infection during the procedure, or postoperative bacteremia of any cause. One reported an aorto-colonic fistula caused by sigmoid diverticulitis [4].

For AEF after EVAR, the mechanism of fistula formation is unclear. One hypothesis is that infection during the procedure or even in a preexisting infected aneurysm can cause intestinal necrosis [5, 6]. There also has been reported a case of fistula formation from graft migration and kinking [7], extensive embolization of the aneurysm sac after EVAR [8], or an endotension that created pressure necrosis in the intestinal wall [9]. In addition, one article reported fistula formation after endovascular treatment of inflammatory aneurysm [10].

Prompt diagnosis of AEF is essential. The prognosis may improve if the diagnosis is not delayed, and immediate treatment is performed [11]. In patients with a history of aortic interventions and ongoing gastrointestinal hemorrhage, the AEF should be recognized as a differential diagnosis until proven otherwise.

Approximately 70% of cases would be preceded by transient and self-limited bleeding (herald bleeding) followed by exsanguinous hemorrhage. Another 30% would present with nonspecific symptoms, such as fever, malaise, and abdominal pain. The time from the first bleeding to diagnosis varies from day to years [12].

In hemodynamically stable patients, endoscopic examination is an investigation of choice, especially in patients with a history of GIB. This can exclude other common causes of bleeding, and 25% of the AEF would be identified [3].

Contrast-enhanced CT is now a valuable tool to detect perigraft infection [13]. It has high sensitivity and specificity of more than 90%. The findings of the AEF from the CT scan include patent air in the aneurysm sac for more than four weeks after the operation, disruption of the aortic wall, and extravasation of contrast in the bowel lumen. Other findings suggest aortic graft infection, for example, loss of fat plane between the aorta and the bowel, focal thickening of the bowel wall, collection of perigraft fluid more than three months after repair, or aortic pseudoaneurysm.

However, combining both diagnostic tools would be essential to increase diagnosis and help further plan treatment, especially in stable patients.

The principles of AEF treatment are the same as for aortic graft infection. The goals are to control hemorrhage, infection, and revascularization to maintain distal perfusion. With conservative management, mortality would be high.

Lately, there are two main modalities of treatment of AEF, including extra-anatomical bypass and in situ reconstruction. For extra-anatomical bypass, a staged procedure can be done with first staged axillo-bifemoral bypass, followed by the removal of the graft and aortic occlusion, or synchronous axillo-bifemoral bypass with the removal of the graft and aortic occlusion, especially in unstable patients.

For in situ reconstructions, a femoral vein [14], a rifampicin-soaked graft [15], a cryopreserved allograft [16], or a silver graft can be used as a conduit after completely debride of all necrotic tissue.

A systemic literature analysis by Bergqvist et al. [11] showed that simultaneous axillo-bifemoral bypass and graft removal had the best outcome. However, as discussed by Batt et al. [17], extra-anatomical bypass and in situ reconstruction seem to have a similar results, with in-hospital mortality at approximately 30–40%, and the risk of recurrent infection can occur during follow-up, regardless of the treatment modality. Long-term follow-up is mandatory.

An endovascular method was used to treat an AEF from a review and a pooled data analysis by Kakkos et al. [2]. This study found that in-hospital mortality was lower with endovascular treatment compared to open surgery, but this benefit was lost during follow-up. On the contrary, performing an endovascular treatment in an emergency operation to control bleeding followed by an open surgery would improve the outcome.

In our study, the etiology of fistula formation was suspected from endovascular repair in an unrecognized infected aneurysm followed by directed pressure necrosis of the sac into the bowel wall. Ischemic colitis from the disruption of IMA after EVAR is least likely because the inferior mesenteric artery (IMA) was quite small at first and all the collateral from superior mesenteric artery (SMA) was patented. AEF was not suspected at first, so elective colonoscopy and CTA were performed. With this stable condition and suspected severe fecal contamination of the aneurysm sac, a stage axillo-bifemoral bypass was performed, followed by graft explantation and Hartmann's procedure.

## 4. Conclusion

Secondary AEF is a rare but lethal disease, often presenting with GIB following aortic surgery. Delayed diagnosis would be fatal or lead to higher morbidity and mortality. Highly suspicious of this condition is a key to prompt investigations. Both endoscopy and contrast-enhanced CT scans would help set the diagnosis. Treatment options need to be carefully considered depending on patients' conditions. Graft explantation and in situ or extra-anatomical bypass would be the best treatment modalities.

## Data Availability

The data used to support the findings of this study are included within the article.
